# Storage-associated shifts in the raw boar seminal microbiome at 17 °C

**DOI:** 10.3389/fvets.2026.1872476

**Published:** 2026-07-28

**Authors:** Nicole Carrasco-Zambrano, Karla Leal, Juan Machuca, Kattia Núñez-Montero, Felipe Pezo, Maria Jose Contreras

**Affiliations:** 1 Instituto de Ciencias Aplicadas, Facultad de Ingeniería, Universidad Autónoma de Chile, Temuco, Chile; 2 Doctorado en Ciencias Biomédicas, Facultad de Ciencias de la Salud, Universidad Autónoma de Chile, Temuco, Chile; 3 Instituto de Ciencias Aplicadas, Facultad de Ciencias de la Salud, Universidad Autónoma de Chile, Santiago, Chile; 4 Facultad de Ciencias Agropecuarias y Medioambiente, Universidad de La Frontera, Temuco, Chile; 5 Centro de Excelencia en Biotecnología Reproductiva (CEBIOR), Facultad de Ciencias Agropecuarias y Medioambiente, Universidad de La Frontera, Temuco, Chile

**Keywords:** 16s sequencing, bacterial community profile, porcine, seminal, seminal preservation, swine

## Abstract

This study addresses the relevance of the bacterial seminal microbiome in boars, considering that bacterial compositional changes during liquid semen storage may affect sperm quality and limit the efficiency of artificial insemination. The objective was to characterize temporal changes in the seminal microbiota composition of raw boar semen incubated at 17 °C for 12 days. For this purpose, six ejaculates from Landrace boars used as artificial insemination donors were analyzed, with samples evaluated on days 1, 4, 8, and 12 through DNA extraction, amplification of the V3–V4 region of the 16S rRNA gene, and Illumina MiSeq sequencing. The results showed a progressive reduction in bacterial richness and diversity during storage, with a transition from an initially heterogeneous community, mainly represented by *Proteobacteria, Firmicutes, Bacteroidota,* and *Actinobacteriota,* toward low-complexity communities relatively dominated by opportunistic taxa such as *Pseudomonas, Escherichia-Shigella, Enterococcus,* and *Bacteroides*. Overall, the study concludes that storage at 17 °C is associated with relative compositional shifts in the raw seminal microbiome toward potentially detrimental bacterial profiles, providing a baseline for understanding the dynamics of the seminal microbiota and supporting the development of more targeted and sustainable antimicrobial strategies for porcine semen preservation.

## 1 Introduction

The study of the boar seminal bacterial-microbiome has emerged as a relevant focus in animal reproduction, given the potential impact of microbial communities on sperm quality and fertility outcomes. Boar ejaculates harbor a diverse microbial population, originating primarily from the male reproductive tract, skin, gastrointestinal tract, and the surrounding environment (1). This microbiota may influence sperm functionality through direct or indirect interactions with sperm and components of the seminal plasma (2–4). Bacterial composition can vary in response to environmental and management factors, and while some microorganisms may exert detrimental effects, others may play a beneficial role in maintaining optimal sperm function (3).

Preservation of boar semen by liquid storage at 15–17 °C is a common practice in commercial swine production systems. However, this process is constrained by bacterial contamination and microbiome changes, which can impair sperm quality during storage and reduce fertility rates (2, 5, 6). Bacteriospermia has been shown to negatively affect motility, viability, and membrane function, in addition to promoting oxidative stress and premature capacitation (5, 7, 8). Despite the prophylactic inclusion of antibiotics in semen extenders, bacterial contamination persists due to the presence of antibiotic-resistant strains (1).

Studies have revealed a negative linear relationship between the abundance of potentially probiotic bacteria (*Lactobacillales*) and harmful bacteria (*Enterobacterales*), with *Lactobacillus* spp. being more prevalent in high-quality ejaculates (3). Differences in specific microbial subsets and their interactions may have a more pronounced effect on sperm motility, morphology, and viability than total bacterial load alone (3, 4). Pathogenic species such as *Pseudomonas aeruginosa, Escherichia coli*, and *Clostridium perfringens* have been associated with reduced motility, alterations in capacitation, and compromised membrane integrity, ultimately impairing fertilizing capacity (5, 7, 8).

Advances in next-generation sequencing (NGS) technologies and metagenomic analyses have enabled comprehensive characterization of the boar seminal microbiome, overcoming the limitations of culture- and PCR-based methods, which often underestimated the presence and diversity of both dominant and low-abundance taxa (9). High-throughput 16S rRNA gene sequencing, combined with bioinformatic analyses, allows precise determination of bacterial composition, diversity indices (3, 9). These approaches have also provided insights into host–microbiome interactions, revealing potential biomarkers and guiding the design of probiotic or antimicrobial strategies to optimize reproductive performance (3, 6, 9). Consequently, understanding the composition and dynamics of the seminal bacterial-microbiota during cooled storage, is essential for developing alternative preservation strategies that maintain sperm quality and reduce antibiotic usage, thereby contributing to more sustainable and efficient swine reproduction systems.

The objective of the present study was to determine changes in seminal bacterial-microbiota composition during incubation at 17 °C for 12 days.

## 2 Materials and methods

### 2.1 Ethical declaration

All experimental procedures and protocols described herein were reviewed and approved by the Scientific Ethics Committee of the Universidad Autónoma de Chile, Temuco, Chile, under authorization code N° BE 01–24 (approval date: March 28, 2024). All procedures were conducted in compliance with the provisions of the Chilean Animal Protection Act (Law N° 20,380). All animals used in this study belonged to a private farm, which authorized access to the animals and the collection of samples for scientific purposes. No additional authorization from the sanitary authority was required to conduct this study.

### 2.2 Reagents

Unless otherwise specified, all reagents were purchased from Sigma (St. Louis, MO, United States). All solutions were prepared using ultrapure water obtained from a Milli-Q Synthesis system (Millipore, Bedford, MA, United States).

### 2.3 Animals

For this assay, six Landrace boars were used. All animals belonged to the same commercial farm and were maintained year-round under confined conditions in boar housing facilities. Their diet was based on grains and a protein supplement provided by canola (*Brassica napus*), triticale (× *Triticosecale Wittmack*), and soybean bran (*Glycine max*). All samples were collected during the summer season, between January and April 2025.

### 2.4 Boar semen collection

Semen samples from sexually mature boars routinely used as donors for artificial insemination (AI) were collected by the gloved-hand technique at a commercial farm located in Victoria, southern Chile. A total of six ejaculates were obtained from different individuals. Raw semen was collected in sterile QuickTip® tubes and transported for one hour to the laboratory in a portable temperature-controlled boar semen cooler maintained at 17 °C for subsequent processing and analysis. Only ejaculates with total sperm motility above 80% and viability above 85% were included in the study.

### 2.5 Experimental design

Raw semen samples were incubated for 12 days at 17 °C. Intermediate evaluations were performed to assess changes in the seminal microbiome. Samples were analyzed at days 1 (immediately after transported), 4, 8, and 12. At each time point, semen was processed as follows: 1 mL of raw semen was transferred into sterile Eppendorf tubes, stored at −80 °C, and subsequently used for DNA extraction and 16S rRNA sequencing.

It should be noted that raw semen was used in this study in order to characterize and evaluate the native seminal microbiome. Conventional storage extenders were not included, as most of them contain antibiotics and would also alter the environmental conditions, potentially leading to modifications in the microbiome composition.

### 2.6 DNA extraction and 16S rRNA region sequencing

A total of 24 samples (6 samples per evaluation time point) were pretreated by centrifugation at 130 *g* at room temperature for 5 min to remove sperm cell, and the supernatant was collected for subsequent DNA extraction using the commercial AccuPrep® Genomic DNA kit (Bioneer, Daejeon, South Korea). DNA concentration from each sample was quantified with the QuantiFluor® ONE dsDNA System (Promega, Madison, WI, United States) following the manufacturer’s instructions s, and integrity of the genomic DNA confirmed by 1% agarose gel electrophoresis. The mean DNA concentration obtained from the samples was 1 ng/μL, and the average A260/A280 ratio was 1.9.

Bacterial 16S rRNA was amplified using a pair of universal primers targeting the V3–V4 regions and the 2 × sparQ HiFi PCR Master Mix (QuantaBio, MA, United States). Following library preparation with Illumina’s 16S Metagenomic Sequencing Library Preparation protocol, paired-end sequencing (2 × 300 bp) of 16S rRNA V3–V4 amplicons was conducted on the Illumina MiSeq platform using the MiSeq Reagent Kit v3. The amplicons were generated using the following primers: Forward primer 5′-CGTCGGCAGCGTCAGATGTGTATAAGAGACAGCCTACGGGNGGCWGCAG and Reverse primer 5′-GTCTCGTGGGCTCGGAGATGTGTATAAGAGACAGGACTACHVGGGTATCTAATCC. The forward and reverse primers included overhang adapter sequences fused to the 16S V3–V4 target region. The sequencing run was normalized to approximately 200,000 reads per sample and included a 30% PhiX spike-in to compensate for low base diversity.

### 2.7 16S rRNA metabarcoding

Raw paired-end data were merged and processed using QIIME2 2024.10. First (10–13), adapters were detected and removed using the Cutadapt 4.9 plugin, filtering sequences based on the 341F-805R primers and retaining only sequences that matched these primers with the --discard-untrimmed flag, for a total of 5,128,281 demultiplexed reads across 24 samples. Then, merging and denoising were carried out using DADA2, setting a manifest file and truncating fragments to 284 nt for forward reads and 280 nt for reverse reads guided by cutadapt-derived mean read lengths, for a total of 3,375 inique amplicom sequence variants (ASVs) across all samples. DADA2 infers amplicon sequence variants (ASVs), which represent exact sequence variants resolved at single-nucleotide resolution. Therefore, all downstream feature counts and diversity analyses were based on ASVs rather than operational taxonomic units (OTUs), which are cluster-based features defined by sequence similarity thresholds. All processing statistics are summarized in Supplementary Table S1. Lastly, taxonomic assignment was performed using a Naive Bayes classifier previously trained on the SILVA 138 reference database (99% OTUs), specifically tailored to the V3-V4 region of the 16S rRNA gene. The SILVA 138 99% OTU reference database was selected because it represents a curated, non-redundant, widely used, and reproducible resource for QIIME 2-based 16S rRNA taxonomic classification. The use of this reference database refers only to the taxonomic reference used for classification and does not imply clustering of the DADA2-derived ASVs generated in this study. Although a full/non-clustered SILVA reference may retain more reference sequences, it requires additional curation and classifier training and does not fully overcome the limited discriminatory power of the V3-V4 region for closely related bacterial species. Therefore, species-level labels were interpreted as putative assignments and used only for descriptive purposes. Post-classification, taxonomic tables were manually curated to remove host-derived, non-bacterial, unassigned, or incomplete taxonomic entries before downstream summaries were generated. The updated list of removed entries, including ASV identifiers, taxonomic assignment, and classifier confidence values, is provided in Supplementary Table S2. In addition, a curated level-7 taxonomic abundance table including the complete lineage from phylum to putative species level, together with raw counts and per-sample relative abundances, is provided as Supplementary Table S3 to allow inspection of intermediate ranks such as class, order, and family. Taxa results and their formats were processed using QIIME2, and relative abundance plus alpha- and beta-diversity analyses were calculated using the MicrobiomeAnalyst webserver. Briefly, data were processed using low-count filtering (minimum count in samples set at 4 and prevalence in samples set at 20%) and low-variance filtering (percentage to remove set at 10% based on the interquartile range). These thresholds correspond to the default MicrobiomeAnalyst marker data profiling settings and were selected *a priori* as a standardized preprocessing step to reduce the influence of sparse, low-prevalence, and low-variance ASVs in downstream comparative analyses (11). Similar MicrobiomeAnalyst-based filtering settings have also been used in previous microbiome studies (14, 15). In the present dataset, the 20% prevalence threshold required an ASV to reach the minimum count in approximately five samples, and the low-variance filter removed only the lowest 10% of features based on the interquartile range. The filtering parameters were not adjusted *post hoc* according to the observed results. Data were then normalized using total sum scaling (TSS). Because no parallel absolute quantification of total bacterial load was performed, all taxonomic results derived from 16S rRNA amplicon sequencing are interpreted as relative compositional changes rather than absolute bacterial proliferation or growth.

### 2.8 Statistical analysis

Alpha-diversity indices, including Chao1, Shannon, Simpson, and observed richness, were calculated for each sample at each storage time point. Since the same samples were evaluated longitudinally during storage, the overall effect of time was assessed for each alpha-diversity index using the Friedman test. This global test was considered the primary inferential analysis because the objective was to evaluate whether alpha diversity changed over time during seminal storage, rather than to test each pairwise time-point comparison as an independent hypothesis. Pairwise comparisons, when shown, were used only to describe the temporal pattern of change and were not interpreted as independent confirmatory outcomes. Beta diversity was assessed using Bray–Curtis dissimilarity and visualized through principal coordinate analysis (PCoA). Differences in microbial community structure among storage time points were evaluated using PERMANOVA with 999 permutations. Because the same samples were evaluated longitudinally across storage days, permutations were restricted by sample identity to account for the repeated-measures structure of the dataset. Given the small sample size per time point and the constrained permutation space associated with the paired design, PERMANOVA results were interpreted cautiously and considered together with the ordination patterns and descriptive taxonomic changes. ASVs rarefaction curves were obtained in order to check for sequencing depth, were built using 20 steps and are presented in Table S1. For the species-level descriptive analysis, sample 1-d4 was not considered a technical failure or a low-depth sample. This sample retained 219,633 non-chimeric reads and 354 ASVs after DADA2, 218,755 reads and 319 ASVs after manual refiltering, and 208,759 reads in the genus-level table. However, only 162 reads were retained in the collapsed species-level table after taxonomic classification and downstream filtering. Therefore, the limitation was specific to species-level taxonomic resolution, and sample 1-d4 was not used for the detailed species-level sample-by-sample description. Raw sequencing data was deposited in the NCBI Sequence Read Archive (SRA) under BioProject accession number PRJNA1320724.

## 3 Results

### 3.1 Phyla level

**Figure 1:**
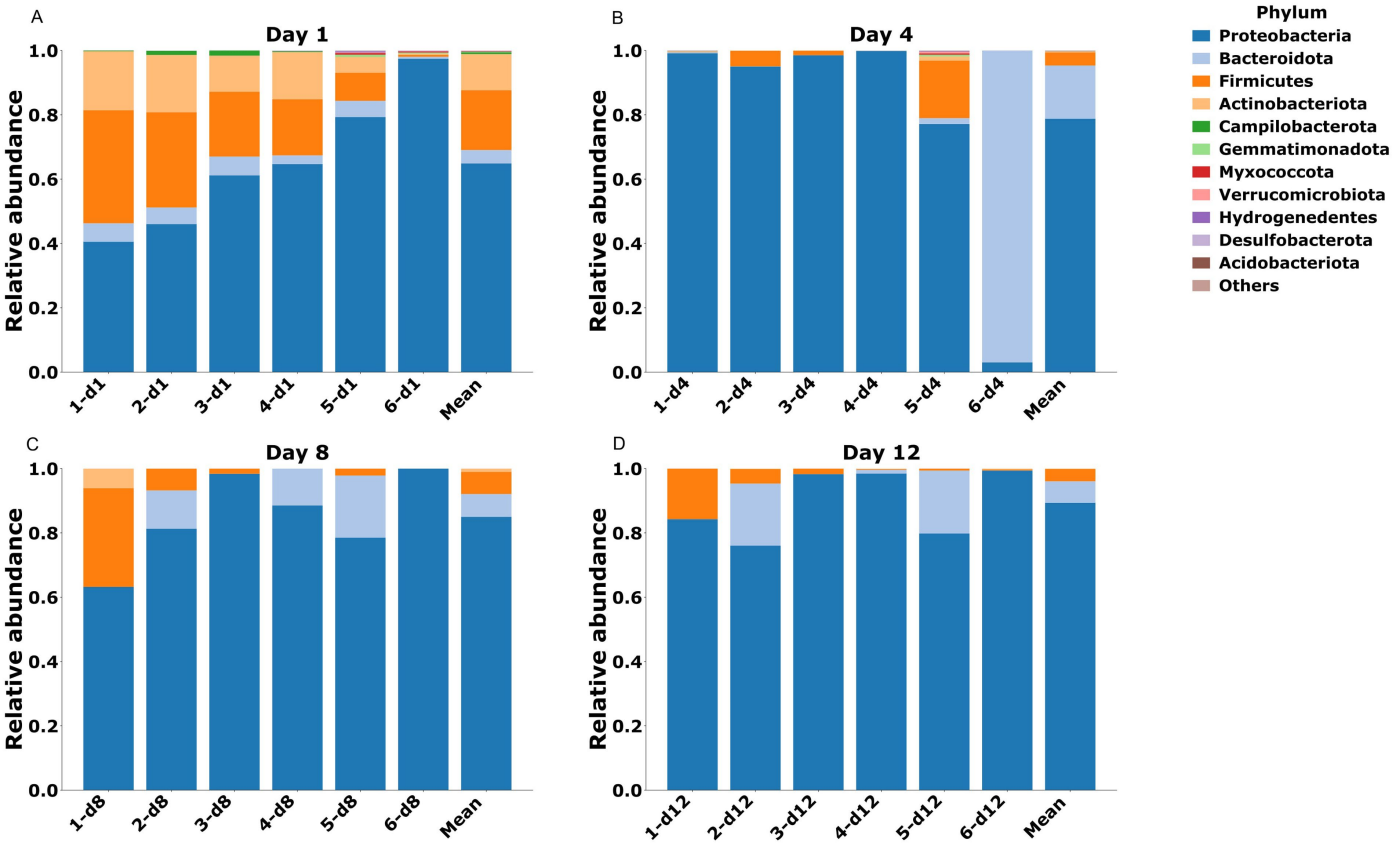
Relative abundance of the top 10 bacterial phyla (>1% relative abundance) in the seminal microbiome of boars incubated at 17 °C. Panel **(A)** Day 1, Panel **(B)** Day 4, Panel **(C)** Day 8, Panel **(D)** Day 12. Sample bars represent individual biological replicates (boars 1–6), and the final bar in each panel represents the mean relative abundance across samples for that storage time point. Colors were kept consistent for each phylum across all panels.

After filtering, a total of 3,229 ASVs were retained for downstream taxonomic and diversity analyses. At the phylum level, the seminal microbiome of boars was relatively dominated throughout storage by *Proteobacteria*, followed by *Firmicutes,* and *Actinobacteriota* (Figure 1). On day 1, diversity was highest, with additional phyla detected in several individuals, including *Campilobacterota, Gemmatimonadota, Myxococcota, Desulfobacterota, Hydrogenedentes,* and *Verrucomicrobiota*, most of them at low relative abundance (<5%). On day 4, the microbiota composition shifted toward higher proportions of *Proteobacteria* and *Bacteroidota*, whereas minor taxa, such as *Campilobacterota*, decreased to the point that they were no longer represented in the graph. In some samples, *Acidobacteriota* also appeared. By days 8 and 12, diversity had markedly declined, and only the four most abundant phyla (*Proteobacteria, Firmicutes, Actinobacteriota, Bacteroidota*) remained consistently detectable across replicates, with *Proteobacteria* showing the highest relative abundance.

### 3.2 Genus level

**Figure 2:**
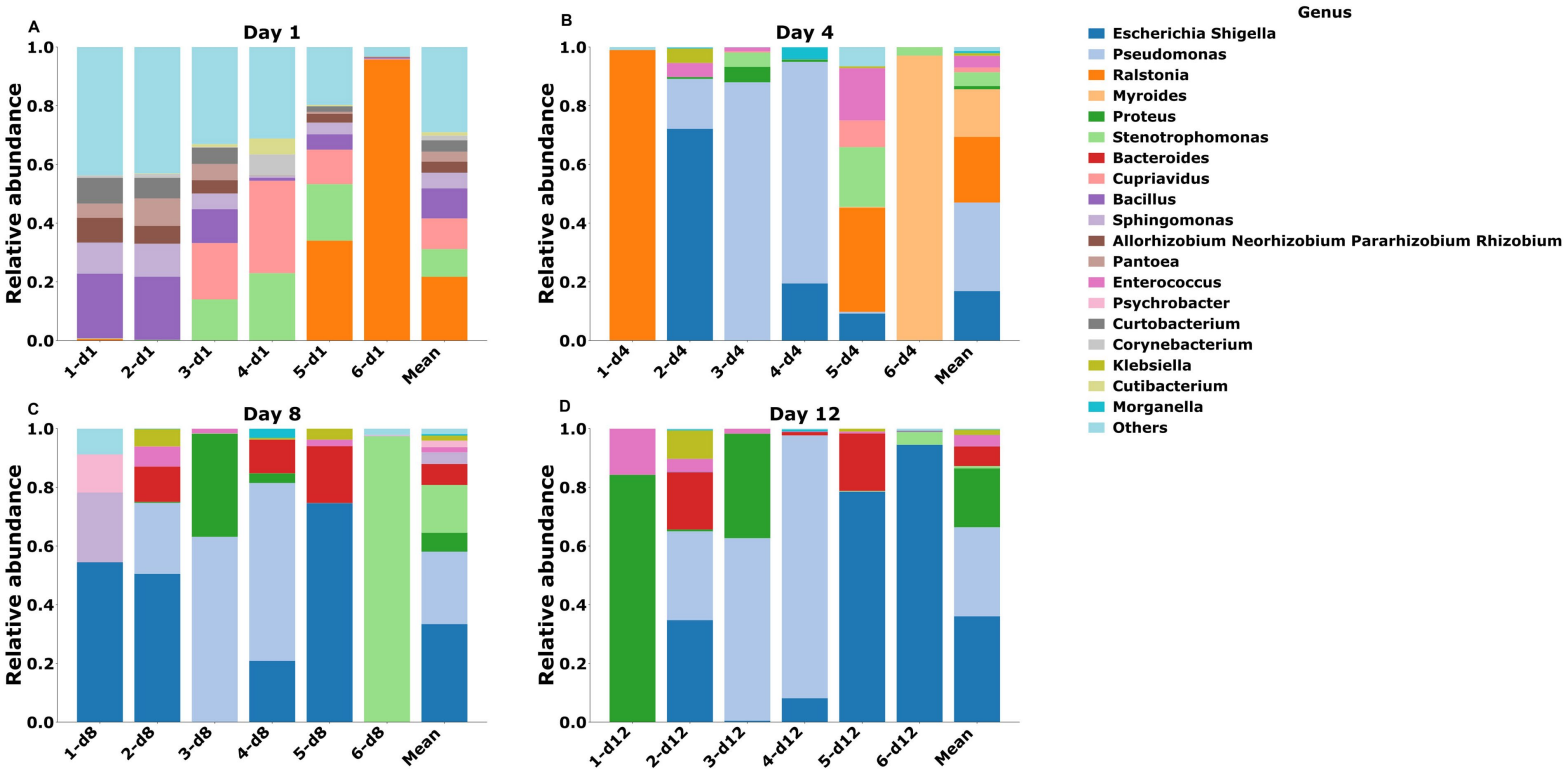
Relative abundance of the top 10 bacterial genera (>1% relative abundance in at least one replicate) in the seminal microbiome of boars incubated at 17 °C. Panel **(A)** Day 1, Panel **(B)** Day 4, Panel **(C)** Day 8, Panel **(D)** Day 12. Sample bars represent individual biological replicates (boars 1–6), and the final bar in each panel represents the mean relative abundance across samples for that storage time point. Colors were kept consistent for each genus across all panels.

Relative abundance analyses at the genus level revealed clear temporal shifts in the seminal microbiome (Figure 2). On day 1 (Figure 2A), the community exhibited high heterogeneity, with multiple genera represented at low relative abundance, including *Cupriavidus, Ralstonia, Bacillus, Sphingomonas,* and *Corynebacterium*.

By day 4 (Figure 2B), however, within-sample diversity decreased and a relative predominance of opportunistic taxa such as *Pseudomonas, Escherichia-Shigella*, and *Myroides* emerged, while genera such as *Ralstonia* and *Stenotrophomonas* remained present but at lower relative abundance. On day 8 (Figure 2C), the community showed a further loss of diversity within the same sample over time and was mainly represented by *Escherichia-Shigella, Pseudomonas, Enterococcus*, and *Stenotrophomonas*. Finally, by day 12 (Figure 2D), richness further declined, and the community was relatively dominated by *Escherichia-Shigella, Pseudomonas*, and *Enterococcus*, with minor contributions from *Proteus* and *Klebsiella*.

### 3.3 Species level

**Figure 3:**
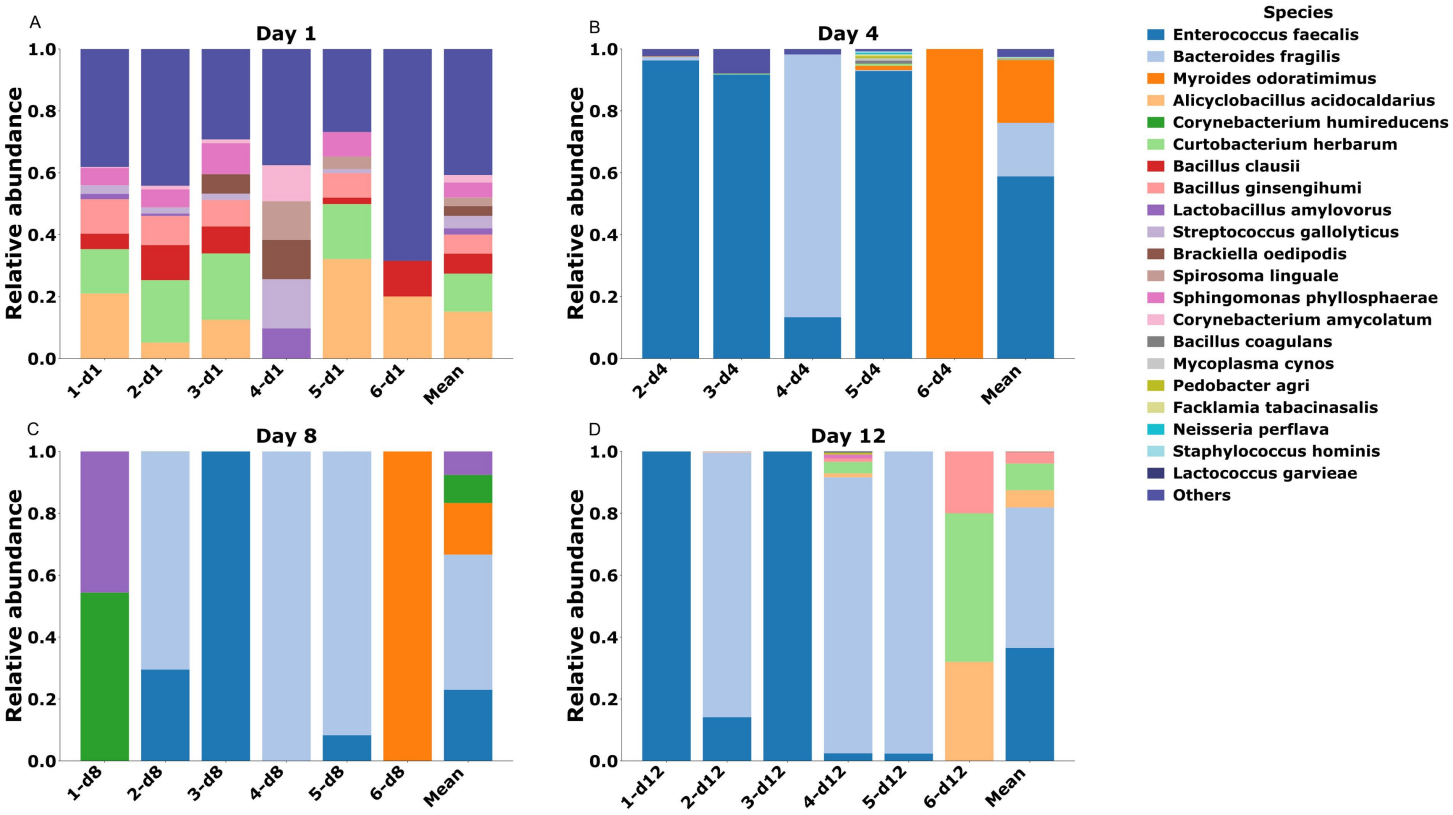
Relative abundance of the top 20 bacterial species (>1% relative abundance in at least one replicate; species-level assignments considered putative and descriptive rather than definitive species identifications) in the seminal microbiome of boars incubated at 17 °C. Panel **(A)** Day 1, Panel **(B)** Day 4, Panel **(C)** Day 8, Panel **(D)** Day 12. Sample bars represent individual biological replicates (boars 1–6), and the final bar in each panel represents the mean relative abundance across samples for that storage time point. Colors were kept consistent for each putative species-level assignment across all panels.

At the species level, the boar seminal microbiome exhibited a marked temporal reduction in diversity, with only a few taxa showing high relative abundance across the incubation period (Figure 3). It is important to note that species-level assignments derived from 16S rRNA sequencing should be considered putative, given the limited resolution of the V3–V4 region and the possibility of misclassification for closely related taxa. Accordingly, species-level labels are presented as descriptive putative assignments and should not be interpreted as definitive species identification. On day 1 (Figures 3A), a heterogeneous community was detected, including *Alicyclobacillus acidocaldarius, Curtobacterium herbarum, Bacillus ginsengihumi, Sphingomonas phyllosphaerae, Streptococcus gallolyticus*, and *Lactobacillus amylovorus*. By day 4 (Figure 3B), diversity had already decreased, and opportunistic species such as *Enterococcus faecalis, Myroides odoratimimus* (only sample 6*)*, and *Bacteroides fragilis* (only sample 4) showed high relative abundance. Notably, strong inter-individual variation was observed, with some samples showing near-total relative abundance of *M. odoratimimus* or *B. fragilis*. On day 8 (Figure 3C), richness declined sharply, with only five species remaining above the detection threshold. At this stage, *B. fragilis* and *E. faecalis* showed the highest relative abundance in most samples, while *Myroides odoratimimus* and *L. amylovorus* persisted in lower proportions. By day 12 (Figure 3D), low-diversity community primarily composed of *B. fragilis* and *E. faecalis*, along with minor contributions from *A. acidocaldarius, Cutibacterium acnes,* and *Lactococcus garvieae*.

When the data were analyzed by sample, evaluating changes over time, the following patterns were observed: In sample 2, day 1 showed a diverse composition, with the presence of *Sphingomonas phyllosphaerae, Bacillus clausii, Bacillus ginsengihumi, Alicyclobacillus acidocaldarius,* and a large relative fraction of “Others.” However, by day 4, an abrupt shift was observed, with an almost exclusive relative dominance of *Enterococcus faecalis*. On day 8, this relative dominance was partially replaced by *Bacteroides fragilis*, although *Enterococcus faecalis* remained present at an important proportion. Finally, by day 12, *Bacteroides fragilis* showed the highest relative abundance, while *Enterococcus faecalis* appeared as a secondary taxon.

In sample 3, day 1 also showed a heterogeneous microbiota, with the presence of *Spirosoma linguale, Bacillus ginsengihumi, Bacillus clausii, Sphingomonas phyllosphaerae, Alicyclobacillus acidocaldarius, Streptococcus gallolyticus*, and other minor taxa. By day 4, the community changed notably, with a clear relative dominance of *Enterococcus faecalis*, accompanied by a low proportion of “Others.” On day 8, the sample became almost completely represented by *Enterococcus faecalis*. Subsequently, by day 12, the sample showed a near-total relative predominance of *Enterococcus faecalis*.

In sample 4, the day 1 community was highly diverse and included *Corynebacterium amycolatum, Brackiella oedipodis, Lactobacillus amylovorus, Spirosoma linguale, Sphingomonas phyllosphaerae,* and other taxa. By day 4, the composition shifted toward a marked relative dominance of *Bacteroides fragilis*, with a low proportion of *Enterococcus faecalis* and other taxa. On day 8, a complete replacement by *Bacteroides fragilis* was observed, reaching near-total relative dominance. By day 12, *Bacteroides fragilis* remained the relatively predominant taxon, although small proportions of other species reappeared, including *Enterococcus faecalis, Bacillus ginsengihumi, Sphingomonas phyllosphaerae,* and *Pedobacter agri.*

In sample 5, day 1 showed a diverse composition, with a relative predominance of “Others,” together with *Alicyclobacillus acidocaldarius, Curtobacterium herbarum, Bacillus ginsengihumi, Sphingomonas phyllosphaerae, Bacillus clausii,* and other low-abundance taxa. By day 4, the community shifted toward a marked relative dominance of *Enterococcus faecalis*, although low proportions of species such as *Bacillus coagulans, Curtobacterium herbarum, Facklamia tabacinasalis, Mycoplasma cynos, Pedobacter agri,* and *Staphylococcus hominis* were still detected. On day 8, *Bacteroides fragilis* showed the highest relative abundance, while *Enterococcus faecalis* remained at low abundance. By day 12, *Bacteroides fragilis* remained the almost exclusively represented taxon in relative abundance, with only a minimal proportion of *Enterococcus faecalis*.

In sample 6, day 1 was characterized by a community composed of “Others,” *Alicyclobacillus acidocaldarius,* and *Bacillus clausii*, among other minor taxa. By day 4, the composition changed completely, with an almost exclusive relative dominance of *Myroides odoratimimus*. On day 8, a new marked shift was observed, with a near-total relative predominance of *Myroides odoratimimus*. Finally, by day 12, the sample showed a more mixed composition, mainly represented by *Curtobacterium herbarum*, together with relevant proportions of *Alicyclobacillus acidocaldarius* and *Bacillus ginsengihumi*.

It should be noted that sample 1-d4 was retained at broader taxonomic levels but was not included in the detailed sample-by-sample species-level description because species-level assignments were poorly retained for this sample after classification and filtering. This was not attributable to low sequencing depth, as sample 1-d4 retained 219,633 non-chimeric reads and 354 ASVs after DADA2 and 208,759 reads at the genus level, mainly assigned to *Ralstonia* (204,503 reads). However, only 162 reads were retained in the collapsed species-level table, supporting a limitation specific to species-level taxonomic resolution.

### 3.4 Diversity analysis

**Figure 4:**
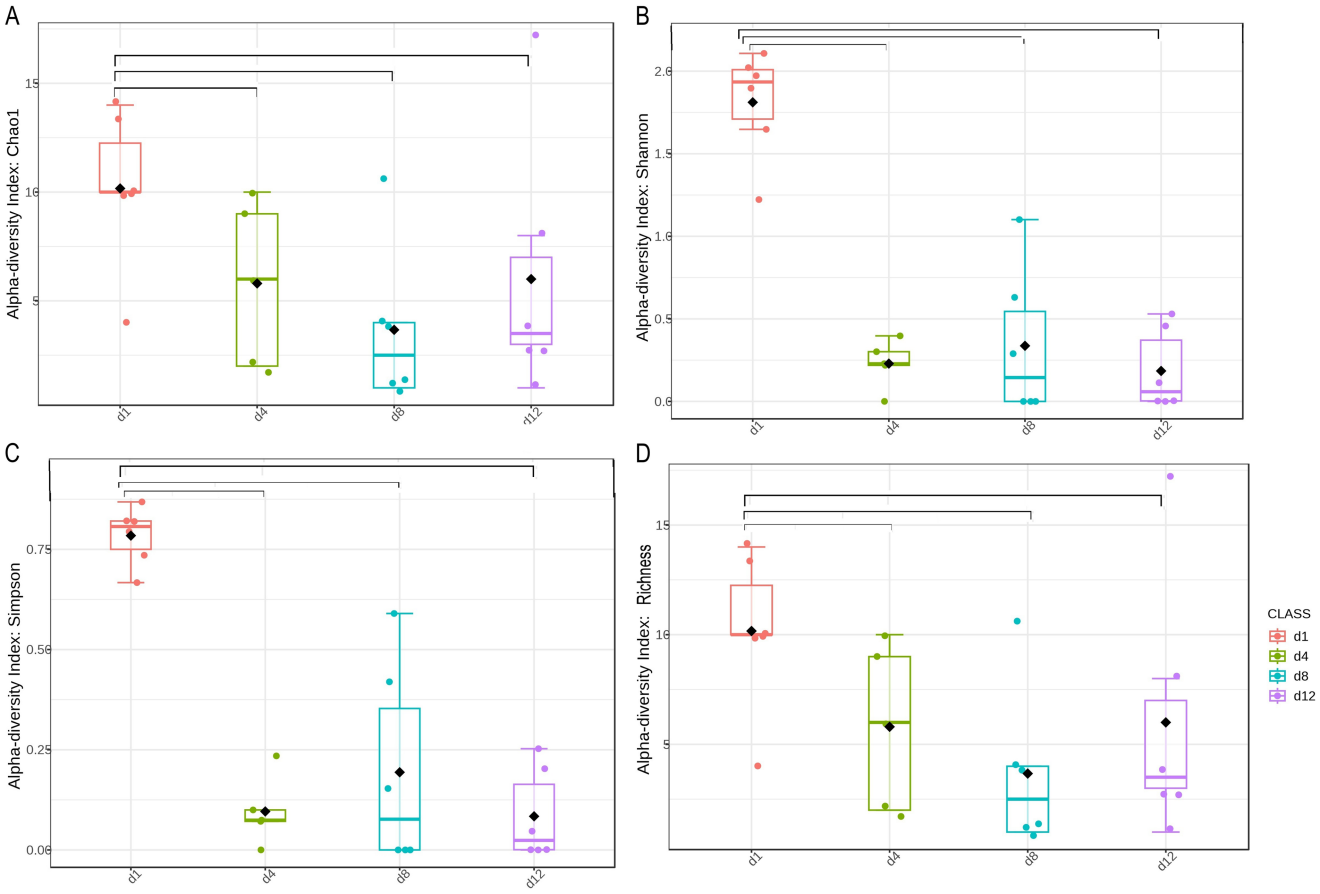
Temporal changes in alpha-diversity indices during seminal storage. Alpha-diversity indices were calculated at four storage time points: day 1 (d1), day 4 (d4), day 8 (d8), and day 12 (d12). Chao1 **(A)**, Shannon **(B)**, Simpson **(C)**, and observed richness **(D)** are shown as boxplots, with individual points representing biological samples and black diamonds indicating mean values. The primary statistical inference was based on the global effect of storage time for each alpha-diversity index, rather than on multiple independent pairwise comparisons among time points. Therefore, pairwise differences are interpreted descriptively and not as confirmatory statistical outcomes. The data show an overall decline in within-sample diversity during storage, with higher alpha diversity at day 1 and reduced values from day 4 onward.

Alpha diversity analyses revealed an overall temporal decline in richness and evenness of the seminal microbiome during storage at 17 °C (Figure 4). The highest values for all indices, including Chao1 (Figure 4A), Shannon (Figure 4B), Simpson (Figure 4C), and observed richness (Figure 4D), were observed on day 1. From day 4 onward, alpha diversity decreased markedly, with lower richness and evenness values throughout the subsequent storage period. By day 8, the indices reached low values, suggesting that the microbial community became relatively dominated by a reduced number of taxa. At day 12, alpha diversity remained reduced, with no evident recovery compared with the initial time point. Overall, these results indicate a temporal reduction in within-sample microbial diversity during storage at 17 °C.

**Figure 5:**
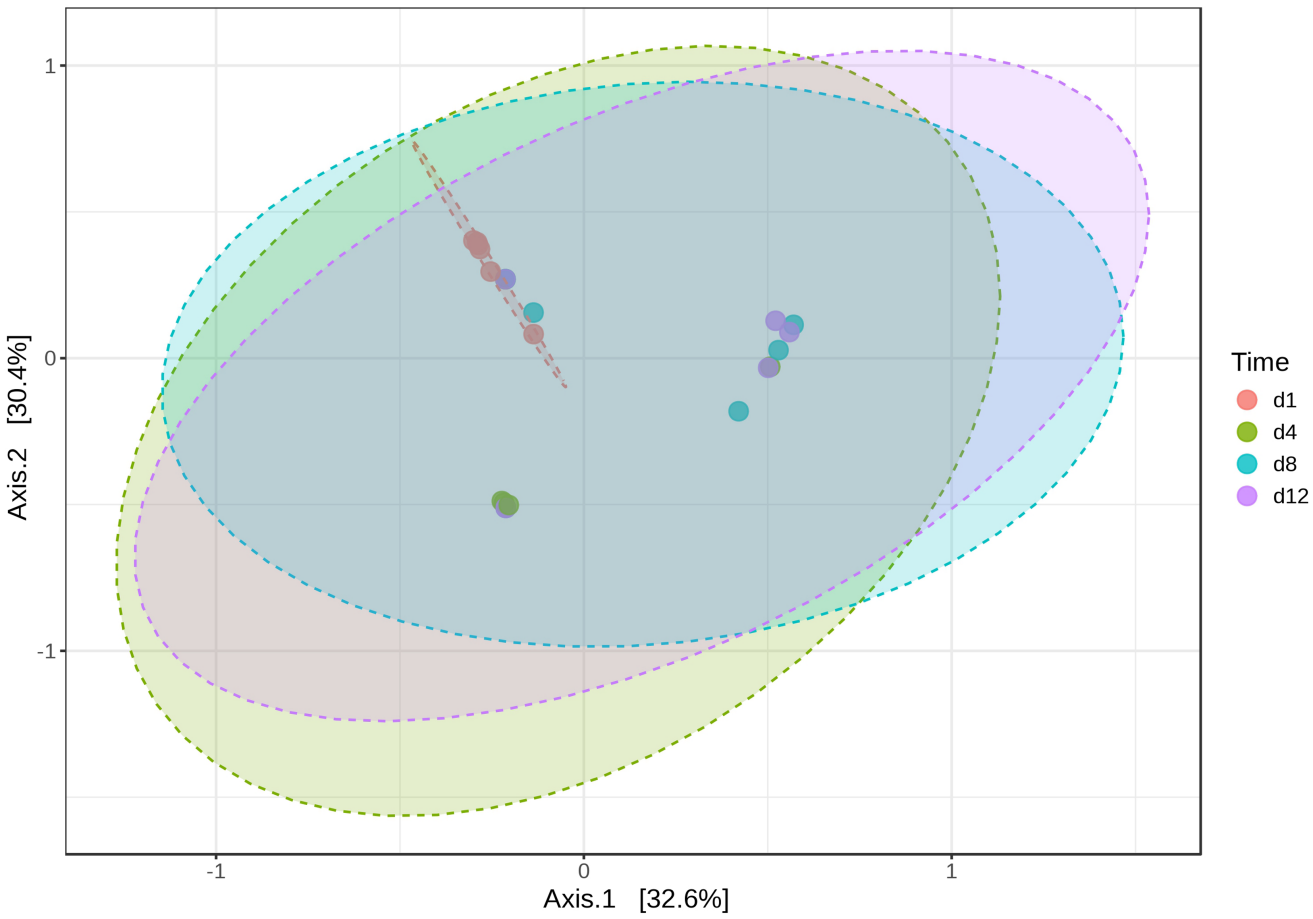
Beta diversity analysis of boar semen bacterial communities at different evaluation times (days 1, 4, 8, and 12). PCoA plots are based on Bray–Curtis distances. Colored ellipses represent 95% confidence intervals for each group.

Beta diversity analyses (Figure 5) suggested temporal restructuring of the seminal microbiome during storage at 17 °C. Principal coordinate analysis (PCoA) based on Bray–Curtis distances showed that the first two axes explained 32.6 and 30.4% of the variance, together accounting for more than 60% of the observed variability. Samples from day 1 tended to cluster closely, indicating a more homogeneous microbial community composition at the initial time point. By days 4 and 8, the dispersion of points increased, suggesting greater inter-sample variability during storage. By day 12, samples appeared to converge again into a more compact cluster that was visually separated from the day 1 group. However, given the small sample size per time point and the restricted permutation space inherent to the longitudinal design, these beta-diversity patterns should be interpreted cautiously and considered together with the descriptive taxonomic changes observed during storage.

## 4 Discussion

This study aimed to characterize the modifications of the seminal microbiome during the incubation of raw semen samples at 17 °C. In the experimental model, the decision was made not to add semen extenders, even though we recognize that their use represents the recommended and commonly applied practice in swine production. This choice was made because the inclusion of extenders, with or without antibiotics, would have altered the natural dynamics of microbiological changes and potentially masked the processes that would naturally occur in seminal samples. We consider this point to be critical and explicitly inform the reader that this methodological decision distinguishes our results from those typically observed under standard swine production conditions where semen extenders are routinely used.

Importantly, this prolonged raw-semen storage model was not intended to reproduce the viability, fertility potential, or commercial shelf-life of extended boar semen doses. Rather, it was designed as a controlled exploratory model to describe the compositional trajectory of the native seminal bacterial community under prolonged cooled-storage stress in the absence of extender-derived nutrients, buffers, antioxidants, or antibiotics. Therefore, the results obtained after extended incubation should be interpreted as changes in the native microbial community under experimental storage stress, and not as evidence of the microbiome composition of reproductively usable semen after 12 days.

In microbiome studies involving native biological samples, the use of media or diluents formulated for eukaryotic cells should be considered a relevant experimental variable, as these media are not chemically neutral for the microbial community present in the sample. Media such as DMEM, RPMI, or MEM are designed to maintain the viability and metabolism of animal cells through the addition of glucose, amino acids, vitamins, salts, buffers, serum, and other supplements. However, these components can modify the original microenvironment of the sample by altering nutrient availability, pH, osmolarity, and redox conditions. Consequently, certain microorganisms may be selectively favored, whereas others may decline, leading to changes in microbiome composition that do not necessarily reflect the native state of the sample. This consideration is particularly important in *in vitro* models of host–microbiome interactions, where culture conditions, oxygen levels, pH, and nutrient availability have been shown to directly influence the stability of microbial communities and the interpretation of results (16–18). Therefore, the incorporation of media or diluents intended for eukaryotic cells may act as an exogenous environmental pressure capable of modifying the original microbiome, and its effects should be recognized and experimentally controlled.

Our study investigated the dynamics of the seminal microbiome during 12 days of incubation. A progressive decline in microbial diversity was observed, accompanied by a shift toward relative dominance of specific taxa. Initially, a diverse community composed mainly of *Proteobacteria, Firmicutes, Bacteroidota,* and *Actinobacteriota* was detected. Over time, the community narrowed and became relatively dominated by *Proteobacteria* and *Bacteroidota* by days 8 and 12. At the genus and species levels, this shift corresponded to the relative enrichment of opportunistic or potentially pathogenic genera such as *Pseudomonas, Escherichia-Shigella, Enterococcus*, and *Bacteroides*. These results suggest that storage conditions are associated with non-uniform compositional changes that favor the relative representation of metabolically versatile and stress-resistant taxa, many of which are associated with pathogenic potential. Storage under suboptimal conditions, including cooled temperatures and absence of extenders or antioxidants, may therefore act as a selective pressure shaping bacterial community composition (2, 5, 19).

Our findings are consistent with previous studies of the boar seminal microbiome. For instance, Zhang et al. (4) reported a diverse bacterial community in fresh boar ejaculates dominated by *Firmicutes, Proteobacteria*, and *Bacteroidota*, resembling the baseline community observed on day 1 in our study. Their work also showed associations between *Pseudomonas* and negative reproductive outcomes, including increased sperm malformation rates, estrus returning rates, stillbirths, and weak offspring. This aligns with our observation that ASVs putatively assigned to *Bacteroides fragilis* and *Enterococcus faecalis* showed relative enrichment during storage and may be associated with semen deterioration. Similarly, Ngo et al. (3) demonstrated that environmental factors influence the porcine seminal microbiome, identifying taxa such as *Globicatella sanguinis* negatively correlated with sperm quality and *Delftia acidovorans* positively correlated. They also reported inverse relationships between dominant groups such as *Lactobacillales* and *Enterobacterales*. These patterns are consistent with our observations that several of these taxa showed increased relative abundance after 8–12 days of incubation at 17 °C, suggesting that environmental stressors such as storage duration, temperature, and lack of extenders promote relative compositional enrichment of potentially detrimental taxa.

The relative enrichment of opportunistic bacterial groups in semen has been associated with impaired sperm function and reduced reproductive performance in previous studies (2, 5, 20). Additionally, Li et al. (21) demonstrated that microbiome composition varies with boar age but consistently includes Firmicutes and Proteobacteria as dominant groups, supporting the diversity observed on day 1.

More broadly, the review by Contreras et al. (1) emphasized that uncontrolled microbial growth in preserved semen represents a major fertility risk by impairing sperm physiology and increasing the probability of pathogen transmission during artificial insemination. Our findings are compatible with this concept, as ASVs putatively assigned to *Enterococcus faecalis* and *Bacteroides fragilis* showed relative dominance during incubation and are known to be associated with oxidative stress, membrane damage, and impaired fertilization capacity. Importantly, clinical evidence from human reproductive medicine also supports the biological plausibility of a detrimental role of opportunistic taxa in semen quality. In a retrospective monocentric study of 359 infertile men, *Enterococcus* spp. were detected in 13.9% of cases, and cumulative pathogen burden was inversely associated with progressive sperm motility, suggesting that the presence and load of urogenital microorganisms may negatively affect sperm function in humans (22).

Alpha-diversity analyses (Chao1, Shannon, Simpson, Richness) confirmed a progressive decline in microbial diversity over time, while beta-diversity analyses revealed temporal clustering patterns. Early samples (day 1) formed a homogeneous cluster, whereas intermediate samples (days 4 and 8) diverged, indicating dynamic compositional succession. By day 12, partial regrouping suggested stabilization of a new microbial community distinct from the baseline. These results indicate that incubation time at 17 °C acts as a strong selective pressure shaping the relative composition of the seminal microbiota (20). The practical relevance of bacterial control during cooled semen storage has been underscored by Luther et al. (23), who demonstrated that uncontrolled bacterial growth during storage at 17 °C was associated with compromised sperm quality, reinforcing the need to monitor and manage microbial dynamics throughout the preservation period.

The relative enrichment of *Pseudomonas, Escherichia-Shigella*, and *Enterococcus* under stressful conditions has also been described in tropical environments, where these genera correlated with reduced sperm motility (3). In our study, the same genera showed high relative abundance by day 12, reinforcing the hypothesis that both environmental and storage conditions act as selective forces promoting compositional seminal dysbiosis. As emphasized by Contreras et al. (1), such microbial imbalances may impair sperm physiology through oxidative stress and increase the risk of pathogen transmission during artificial insemination.

From a management perspective, control of the seminal microbiome has practical implications. Studies evaluating storage at 5 °C have demonstrated that lower temperatures reduce bacterial growth and may decrease the need for antibiotics without compromising fertility (24, 25). However, excessive cooling increases the risk of chilling injury, indicating that an optimal balance between microbial control and sperm preservation must be achieved.

A main limitation of this study is that the ejaculates were obtained from a single farm, from animals of a single breed, and under one climatic zone. Therefore, although the results describe the temporal dynamics of the boar seminal microbiome under the evaluated conditions, their extrapolation to other production systems, breeds, reproductive management practices, or climatic environments should be made with caution. In this regard, future studies should include different farms, genetic lines, and environmental conditions to strengthen the external validity of these findings.

The 16S rRNA approach provides limited taxonomic resolution, and relative abundance data do not reflect bacterial viability, activity, or absolute bacterial load. In addition, taxonomic assignment was performed using a classifier trained on the SILVA 138 99% OTU reference database. This database was selected as a curated, non-redundant, widely used, and reproducible resource for QIIME 2-based 16S rRNA classification. It was used only for reference-based classification and did not collapse the DADA2-derived ASVs, but its clustered structure may constrain species-level resolution. Although a full/non-clustered SILVA reference may retain more reference sequences, it would not fully resolve the marker-level limitation of the V3–V4 region for discriminating closely related species. In addition, because total 16S rRNA gene copy number per millilitre was not quantified by qPCR or another absolute method, the observed increases in relative abundance cannot distinguish true absolute growth of specific taxa from the decline of other community members, or both processes simultaneously. Future studies integrating absolute bacterial quantification, metagenomics, metatranscriptomics, and culture-based approaches will be necessary to clarify the functional roles of specific taxa and their interactions with sperm cells. The study of the native microbiome of porcine seminal samples during 12 days of storage provides a baseline for identifying which bacterial taxa show the highest relative abundance, persistence, or potential relevance during semen preservation for reproductive purposes. This information is important because bacteriospermia can affect sperm quality and reduce the shelf life of seminal doses, while the routine use of antibiotics in semen extenders has been questioned due to its potential contribution to antimicrobial resistance (1). However, one limitation of this approach is that, under commercial conditions, porcine semen is normally stored diluted in commercial extenders, which modify the seminal environment through the addition of nutrients, buffers, salts, and antibiotics. Therefore, the analysis of native semen is not intended to fully reproduce field conditions, but rather to provide foundational knowledge about the bacterial taxa that become relatively predominant during storage. This information may guide the future formulation of semen extenders with more specific antimicrobial strategies targeting the bacterial taxa that are truly relevant, thereby promoting focused antibiotic therapy and reducing the prophylactic use of broad-spectrum antimicrobials (23). This approach is aligned with current efforts to reduce antibiotic use in porcine artificial insemination and to develop more sustainable reproductive systems.

## 5 Conclusion

This study demonstrates that storage of raw boar semen at 17 °C induces marked temporal shifts in the seminal bacterial microbiome, characterized by a progressive loss of microbial richness and diversity and the selection of low-complexity communities relatively dominated by opportunistic taxa. Although the fresh seminal microbiome initially showed a heterogeneous and diverse bacterial profile, prolonged incubation was associated with increased relative abundance of genera such as *Pseudomonas, Escherichia-Shigella, Enterococcus,* and *Bacteroides*, as well as putative species-level assignments, including *Enterococcus faecalis* and *Bacteroides fragilis*. These findings suggest that cooled storage acts as a strong selective pressure associated with compositional reshaping of the native seminal microbiota toward potentially detrimental bacterial profiles. Overall, these findings highlight the importance of monitoring the seminal microbiome during semen preservation and support the development of targeted antimicrobial or microbiome-modulating strategies aimed at preserving sperm quality while reducing reliance on broad-spectrum antibiotics in swine artificial insemination systems.

## Data Availability

The datasets generated for this study can be found in the NCBI Bioproject ID: PRJNA1320724. [https://www.ncbi.nlm.nih.gov/bioproject/PRJNA1320724/].

## Supplementary material

SUPPLEMENTARY FIGURE 1 Rarefaction curves of observed ASVs per sample. Curves were generated in Microbiome Analyst using 20 steps to evaluate within-sample richness. Except for sample 1-d8, most curves approached an asymptotic plateau, indicating sufficient sequencing depth for diversity analyses.

SUPPLEMENTARY TABLE S1 Per-sample QC, denoising summary and ASV counts after filtering. On average, ~300,000 read pairs were obtained per sample. Adapter/primer mismatch was generally low; the main exception was sample 1-d1, where ~44% of pairs lacked the expected adapters and were discarded. The highest ASV richness observed was 660 unique sequences, with day-1 samples showing similarly high values and a gradual decline in ASV counts at later time points.

SUPPLEMENTARY TABLE S2 List of removed OTUs with corresponding taxonomy. List of removed OTUs with corresponding taxonomy. Discarded OTU IDs (SILVA 138) with their taxonomy and classifier confidence are tabulated. OTUs were excluded if classified as mitochondria, chloroplast, Eukaryota, or if labels were incomplete/unassigned. In total, 146 taxa were removed prior to abundance and diversity analyses.

SUPPLEMENTARY TABLE S3 Curated level-7 taxonomic abundance table including complete bacterial lineages, raw counts, and per-sample relative abundances.
