# Supplementary material for: Storage-associated shifts in the raw boar seminal microbiome at 17 °C
Source: Front Vet Sci. 2026 Jul 28;13:1872476. doi: 10.3389/fvets.2026.1872476 (PMC13459440; doi:10.3389/fvets.2026.1872476)
Supplement: Supplementary file 1 [file Image_1.pdf]

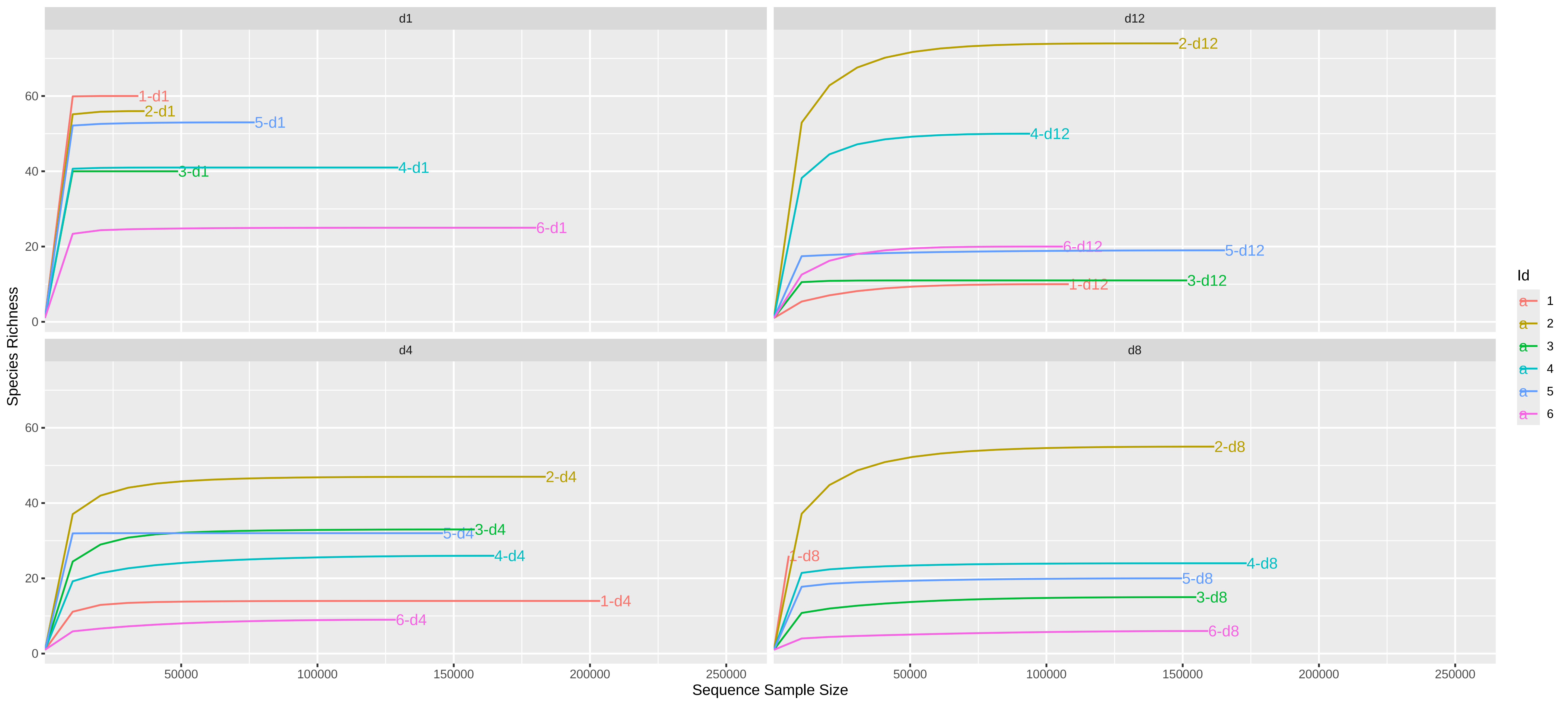
d1
d12
d4
d8
Species Richness
Sequence Sample Size
Id
1
2
3
4
5
6
1-d1
2-d1
5-d1
4-d1
3-d1
6-d1
2-d12
4-d12
6-d12
5-d12
3-d12
1-d12
2-d4
3-d4
5-d4
4-d4
1-d4
6-d4
2-d8
1-d8
4-d8
5-d8
3-d8
6-d8
0
20
40
60
50000
100000
150000
200000
250000
